# Optimizing lower limb rehabilitation: the intersection of machine learning and rehabilitative robotics

**DOI:** 10.3389/fresc.2024.1246773

**Published:** 2024-01-26

**Authors:** Xiaoqian Zhang, Xiyin Rong, Hanwen Luo

**Affiliations:** ^1^ Department of Joint Osteopathy, Liuzhou Worker’s Hospital, Liuzhou, Guangxi, China; ^2^ Center of Neuroengineering, Shenzhen Institute of Advanced Technology, Chinese Academy of Sciences, Shenzhen, China; ^3^ Patent Department, Johnson Intellectual Property Agency, Shenzhen, China

**Keywords:** lower limb rehabilitation, machine learning, rehabilitation robotics, personalized therapy, algorithm challenges

## Abstract

Lower limb rehabilitation is essential for recovery post-injury, stroke, or surgery, improving functional mobility and quality of life. Traditional therapy, dependent on therapists' expertise, faces challenges that are addressed by rehabilitation robotics. In the domain of lower limb rehabilitation, machine learning is progressively manifesting its capabilities in high personalization and data-driven approaches, gradually transforming methods of optimizing treatment protocols and predicting rehabilitation outcomes. However, this evolution faces obstacles, including model interpretability, economic hurdles, and regulatory constraints. This review explores the synergy between machine learning and robotic-assisted lower limb rehabilitation, summarizing scientific literature and highlighting various models, data, and domains. Challenges are critically addressed, and future directions proposed for more effective clinical integration. Emphasis is placed on upcoming applications such as Virtual Reality and the potential of deep learning in refining rehabilitation training. This examination aims to provide insights into the evolving landscape, spotlighting the potential of machine learning in rehabilitation robotics and encouraging balanced exploration of current challenges and future opportunities.

## 1 Introduction

Lower limb rehabilitation is vital for recovery after injury, stroke, or surgery, impacting mobility and quality of life (1). Traditional techniques mainly involved physical therapy, including manual exercises. These methods, although effective, had limitations such as dependence on therapist skills and being physically demanding (2).

Technological advances led to mechanized methods like treadmill training with body weight support and robot-assisted therapy. Body-weight-supported treadmill rehabilitation has demonstrated efficacy in stroke recovery (3). Robot-assisted therapy, in particular, has proven to improve walking speed and distance in patients with stroke compared to conventional therapy (4). Nevertheless, they also present challenges such as high costs (5).

Recently, machine learning has shown potential in reducing costs in lower limb rehabilitation, offering solutions that can be managed with less dependency on highly skilled therapists. This cost-efficiency aspect, highlighted in studies such as Das et al. (6), aligns with the growing need for more accessible rehabilitation options (6). Following this, machine learning also plays a pivotal role in optimizing personalized rehabilitation protocols and predict patient outcomes (7). However, challenges like data privacy, the need for extensive datasets, and model interpretability restrict its clinical application (8).

This review explores the potential of integrating machine learning into lower limb rehabilitation, focusing on its enhancement of rehabilitation robotics. It examines applications in optimizing personalized protocols, predicting recovery, and real-time feedback, and discuss challenges in model interpretability, economics, and regulation. Furthermore, it outlines future prospects, such as incorporating deep learning and virtual reality to enhance rehabilitation training. Selected articles are categorized in Table 1. This review emphasizes balancing current challenges with future opportunities in the evolving field of lower limb rehabilitation.

**Table 1:** Summary of selected scientific literature on machine learning in lower limb rehabilitation robotics.

Theme	Category of models/methods	Application areas	Input data	References
Powered exoskeletons	Exoskeleton design	Restoring walking in paraplegics	Ambulatory function assessment data	(9)
	Exoskeleton design and control	Assisted walking and gait re-education training	Gait speed, motion pattern data	(4, 10–12)
Robotic technology	Rehabilitation robot design and control	Post-Stroke rehabilitation	Limb and gait neurophysiological data.	(13, 14)
Dynamics	Robotics dynamics optimization and parameter estimation	Robotics	User intent, joint angle predictions, action recognition, and sensor feedback.	(15–17)
Rehabilitation therapy	Rehabilitation assessment and intervention methods	Stroke rehabilitation and limb amputation rehabilitation	rs-fMRI analysis, adaptive control parameters, force and CoP data.	(7, 18–20)
Real-time monitoring	Real-time biosensor systems	Sports rehabilitation and fitness	Gait data from IMU and motion capture systems.	(21)

## 2 Robotics, machine learning, and personalized rehabilitation

Robotic-assisted rehabilitation, enhanced by machine learning algorithms, addresses patients' unique needs and conditions to increase lower limb rehabilitation efficiency. This intersection of robotics and machine learning results in highly individualized, adaptive treatment plans, real-time feedback, and sophisticated data analysis (14).

### 2.1 Customizing rehabilitation plans with advanced machine learning

#### 2.1.1 Deep neural networks (DNNs)

Within the realm of robotic-assisted rehabilitation, Deep Neural Networks (DNNs) play a pivotal role in deciphering intricate biomechanical data and movement patterns captured through devices like gait analysis tools. These networks excel in identifying subtle alterations in gait, allowing the rehabilitation system to adapt interventions precisely to each patient's unique requirements (21, 22). Notably, DNNs have been effectively employed in tailoring robot-assisted exercises based on the severity of paralysis, leading to more effective and personalized therapy outcomes (23). For instance, Just as presented in the study by Esquenazi et al. (9), their research used exoskeletal robotics to stimulate walking rehabilitation of paraplegic patients (9). patients with varying mobility levels receive exercises tailored to their abilities, ensuring optimal progress.

#### 2.1.2 Strategic adjustments via decision trees

In contrast, Decision Trees emerge as a strategic asset for predicting necessary adaptations in rehabilitation strategies (24). By considering factors such as heart rate, balance, and other biomechanical data, these trees forecast improvements in motor skills during rehabilitation, guiding informed modifications to treatment plans (25). Decision Trees prove invaluable in discerning the most suitable robot-assisted interventions for specific patient groups, such as individuals with varying degrees of upper limb impairment due to chronic stroke (13, 26). This underscores the critical role of Decision Trees in shaping tailored and effective rehabilitation paths.

### 2.2 Monitoring progress and predicting recovery

A successful neuro-rehabilitation journey necessitates precise progress monitoring and anticipatory capabilities, both of which can be facilitated through machine learning techniques (27). For instance, the Lokomat gait trainer deploys Reinforcement Learning (RL) to dynamically calibrate the level of assistance provided to patients, ensuring an appropriate challenge level that aids in fostering recovery (28). Acknowledging that a singular assistance mode may not suffice for diverse patient needs, Keller, Rauter, and Riener devised the As Needed (AAN) path controller. This innovation tailors the assistance mode to the patient's condition, adding a layer of adaptability to conventional robot-assisted systems (12). In addition to real-time tracking, accurate predictions about rehabilitation progress and the optimization of therapeutic approaches heavily depend on historical data. Specialized deep learning models tailored for time series data, including Recurrent Neural Networks (RNNs) and Long Short-Term Memory (LSTM) networks, offer powerful predictive capabilities (11). An illustration of this lies in the ReWalk robot exoskeleton system, which employs supervised learning to fine-tune assistance intensity based on previous therapy data, ensuring a consistent trajectory of rehabilitation progress (29, 30). In essence, the synergy between advanced machine learning models like Deep Neural Networks, Decision Trees, Reinforcement Learning, and time-sensitive models such as RNNs and LSTMs is pivotal in revolutionizing the landscape of robotic-assisted rehabilitation. These models collectively enable meticulous analysis of biometric data, tailoring of treatment plans, and precise tracking of recovery progress, culminating in highly effective and personalized therapeutic interventions for patients.

## 3 Comparing with conventional approaches

Exploring machine learning's role in lower limb rehabilitation, it's essential to contrast it with traditional methods. Traditional approaches have relied on manual or mechanical techniques, but machine learning introduces enhanced capabilities, significantly enriching rehabilitation processes (18). Machine learning, especially in lower limb rehabilitation robotics (MLLERR), excels in action intention recognition and gait analysis. An example is the innovative use of a Bayesian optimization algorithm, adeptly bridging the gap between virtual simulations and real-world application (17, 31). This approach was demonstrated in a study using offline data mining and machine learning to predict and evaluate movements in human exoskeleton systems (16).

In lower limb exoskeletons, a standout is the advanced squatting action controller. It showed remarkable accuracy in joint angle tracking, maintaining performance even under a 100N external disturbance force, highlighting its reliability (19). Similarly, a human motion recognition system achieved an impressive 99.61% accuracy in identifying various actions, including falls (17).

Machine learning notably impacts lower limb rehabilitation, enhancing motion controller and exoskeleton performance (15). It can significantly reduce knee extension muscle load during assisted squats, demonstrating its potential in easing physical strain (32, 33). Though still emerging in prosthetics and orthotics, machine learning promises more efficient management of prostheses and orthoses (34).

Table 2 delineates machine learning's benefits over traditional methods in this field. In essence, while traditional techniques form the bedrock of lower limb rehabilitation, machine learning's integration promises increased accuracy, adaptability, and tailored care, addressing the complexities of human gait. Its evolving role is poised to become increasingly pivotal in rehabilitation (35).

**Table 2:** Comparative advantages of machine learning in lower limb rehabilitation.

Advantage	Description	References
Addressing “sim-to-real”	Multi-strategy Bayesian optimization algorithm based on virtual motion parameters effectively addressed sim-to-real transfer	(31)
Enhanced accuracy	Machine learning-based recognition of actions such as falling, walking, and turning achieved 99.61% accuracy	(17)
Strengthened exoskeleton controller	Developed squatting action controller exhibited average joint angle tracking error of only 1.22 degrees	(19)
Reduced physical strain	Machine learning-driven exoskeleton lowered extension muscle load by up to 87.5% during 50% body joint torque-assisted squats	(32)
Increased adaptability	Machine learning allowed for the design of complex exoskeletons that imitate human ankle motion range, significantly improving walking and balance capabilities	(29)

A clinically-based research is needed to maximise lower limb rehabilitation efficacy utilising machine learning and rehabilitative robots. This goal can only be achieved this way. To complete the task, this is the only method. In clinical rehabilitation, Erdaş, Sümer and Kibaroğlu found that a deep learning model, specifically a convolutional neural network (CNN), is better at predicting stroke patients' gait patterns. CNN predicted gait patterns more accurately which was shown by the CNN's superior accuracy than other models. Convolutional neural networks excel at this. This is because the model outperformed conventional methods because this model outperformed established statistical methodologies (36). However, Katakam et al. found that a random forest model predicts functional gains in orthopaedic patients equally well. Closer inspection revealed that these people have improved their functioning. This measure was taken to anticipate functional capability enhancements (37). Machine learning models' accuracy and efficacy are crucial to their usability. This is one of the most important factors in using these models in therapy. Deep neural networks, random forests, and support vector machines have variable degrees of success in predicting rehabilitation results across research (38). This must be considered to ensure accuracy. Remembering this information is crucial for this kind of challenges. Selecting the right model requires careful consideration of the clinical scenario's environmental factors. This is because clinical situations include many environmental factors. If this approach is being followed, it will make sure the model works well and is relevant to the rehabilitation situation being reviewed. Applying machine learning to real-world situations is a major challenge to smooth integration into clinical operations. This is a crucial hurdle to overcome in order to complete the job. Vollmer et al. highlighted the importance of researching both the accuracy of machine learning applications and the practical challenges of implementing them in clinical settings. This research should address the issues raised. This research is necessary to highlight the problem's severity. Performing a comparative study was particularly harder because the patients were different and there were several rehabilitation methods (39). Some researcher recommended adapting machine learning models to clinical situations. Models may work effectively in controlled situations but struggle to meet patient needs in clinical practice. This is because clinical practice feels more natural (40). These models had several flaws, as indicated. It is crucial to have multi-modal data sources. It showed that biomechanical data and patient-reported outcomes can predict knee rehabilitation patients' functional outcomes (41). This was shown by researchers. This method was used to predict functional application results. This comprehensive strategy, which improves machine learning application efficiency, allows for specialized insights into rehabilitation sequences. Evaluation and explanation are crucial when implementing machine learning models in healthcare. Models that provide explicit explanations of predictions and such explanations are crucial, since such models supply both explanations. These models help doctors build trust with patients and make informed judgements. Together with machine insights, this is done (42). A thorough comparison of machine learning models in clinical rehabilitation data is needed to optimize lower limb rehabilitation. This research is necessary for progress. This requirement must be met to achieve desired results. This will enable therapy efficacy improvements, which is vital. Researchers can contribute to the machine learning-rehabilitative robotics conversation by addressing efficacy, accuracy, real-world applicability, patient heterogeneity, multi-modal data integration, and interpretability. Since these fields intersect, the conversation is happening there. This is because the discourse is happening where these two fields intersect. Due to this situation, they can shape the future of customized and effective lower limb rehabilitation treatments.

## 4 Challenges and future directions

### 4.1 Model interpretability

Interpretability in machine learning models is crucial, especially in lower limb rehabilitation (43). This refers to the degree to which a human can comprehend the decision-making process of a machine learning model (44). In the medical domain, and particularly in lower limb rehabilitation, understanding the rationale behind a model's recommendations is paramount. This is because the stakes are high, and decisions directly impact a patient's health and quality of life.

Lack of interpretability can lead to a variety of issues. First, if a model's decisions cannot be understood or explained, it can lead to mistrust from patients, which can hinder their rehabilitation progress (45). For example, if a robotic-assisted therapy device, guided by a machine learning algorithm, recommends a specific exercise regimen, the patient might be hesitant to comply if the rationale is not clear. Second, uninterpretable models can be dangerous in healthcare settings as incorrect interpretation can lead to inappropriate treatment decisions (46). Consider a scenario where an algorithm predicts a slow recovery trajectory; if clinicians cannot understand how this prediction was derived, they might make ill-informed adjustments to the therapy. Lastly, lack of interpretability can pose a challenge in terms of regulatory compliance, as healthcare is a highly regulated field that often requires an explanation of treatment decisions (47–49).

Strategies to improve model interpretability are, therefore, critical. One approach is to use simpler, easily interpretable models like linear regression or decision trees (44). However, this may compromise prediction accuracy. Alternatively, post-hoc interpretation methods like LIME or SHAP can be used to explain complex models (50). These methods approximate the model's decisions locally using a simpler model. Another strategy is to incorporate domain knowledge in model development. This can help in creating models that align with well-understood clinical principles, thereby improving interpretability (51).

### 4.2 Implementation and acceptance: economic, regulatory, and practical challenges

AI-assisted health and medical devices face significant challenges, particularly in economic, regulatory, and practical contexts. Lower limb rehabilitation robots require substantial investment, posing accessibility issues for patients, especially in resource-constrained scenarios (52). Although research suggests that eventual cost savings could arise from improved patient outcomes and operational efficiency, the initial investment required for rehabilitation robots is a substantial hurdle. It's crucial to acknowledge and address the substantial upfront expenses by devising approaches to enhance affordability and accessibility, thereby promoting broader adoption (53). Also, strategies to minimize costs and enhance coverage, such as improving technical designs, optimizing manufacturing processes, and implementing effective rehabilitation strategies, should be prioritized in future studies (54).

The medical sector operates within strict regulatory frameworks aimed at safeguarding patient well-being and treatment effectiveness. Navigating the intricate journey from experimental prototypes to commercially viable AI-assisted rehabilitation devices involves traversing complex regulatory pathways. Demonstrating compliance with stringent medical device regulations, including obtaining approvals from regulatory bodies, entails a time-consuming and resource-intensive endeavor. Collaborative efforts among researchers, manufacturers, and regulatory authorities are indispensable to expedite and streamline this transitional process (55).

Additionally, AI-assisted rehabilitation faces implementation challenges. Rehabilitation robots require specialized knowledge, posing a significant hurdle for healthcare professionals (56, 57). The adaptation of new rehabilitation techniques may pose challenges due to changes required in existing treatment plans and demands on the implementation environment (10, 58, 59).

High equipment costs and maintenance needs can also reduce acceptance (20, 60). However, the potential benefits of AI and rehabilitation robots in lower limb rehabilitation are compelling (61, 62). Personalized rehabilitation treatments provided by robots like Amadeo offer improved rehabilitation efficiency (63).

The triumph of AI-assisted lower limb rehabilitation hinges upon securing the trust and active participation of both patients and clinicians. Patients must feel assured and grasp the advantages of these technologies in their recovery journey. Skepticism or lack of comprehension concerning AI-driven recommendations can impede patient adherence. Moreover, clinicians' endorsement of AI assistance is pivotal, as they play a crucial role in treatment determinations. Fostering alignment between technological progress and the preferences of patients and clinicians necessitates adept communication and comprehensive education (64).

### 4.3 Future directions

One of the most important areas to examine in the future of this discipline is new applications and machine learning techniques in therapeutic rehabilitation. Even though the research has illuminated current methods, there is still room for creative approaches. One of the significant field of the research is to find the small solutions that may enhance the treatment outcomes. This objective of this research can be achieved with the help of clinical rehabilitation as well as machine learning research. Rehabilitation programs may benefit from a more personalized approach that tailor's therapy to patients' needs. This strategy tailor's therapy to each patient's qualities and reactions. Investigating machine learning model scalability and generalizability across clinical populations may lead to further research. Therefore, it is crucial to understand how these models function across a wide range of demographic groupings to ensure their efficacy in various settings. This personalized method makes machine learning deployment more flexible. Through equity, this technique helps create accessible and equitable rehabilitation solutions for all. Real-time monitoring and feedback systems also make investigating extra study intriguing. Researchers can create machine learning algorithms that dynamically tailor rehabilitation therapy to patient needs. These models use wearable sensors and continuous data streams. This is achievable because these models learn from their own experiences. This proactive and adaptive strategy could revolutionize rehabilitation by providing timely and individualized therapies. These interventions help people recover from injuries during the dynamic rehabilitation process. To make matters worse, one of the best areas of future research is the ethical implications of machine learning in therapeutic rehabilitation. One of the most important subjects to study. This is one of the most important topics of research. The importance of addressing data privacy, authorization, and algorithmic bias is unquestionable given the growing adoption of these technologies in healthcare. Researchers can help rehabilitation settings use machine learning ethically by investigating and addressing ethical issues. Ethical assessment and minimization can achieve this. This technique will help patients trust doctors.

## 5 Conclusion

This extensive analysis of machine learning in clinical rehabilitation shows the many ways these technologies can improve patient outcomes. The research shows how machine learning has been used for targeted treatments, predictive modelling, and result evaluations. The ever-changing clinical rehabilitation environment, which emphasizes customized and flexible therapies, requires acknowledging changes. The appraisal of future research shows how important it is to comprehend clinical rehabilitation and machine learning. The junction is where the two fields intersect, explaining this disparity. The current trend involves a paradigm shift towards tailored medicines that consider each patient's uniqueness. This proposal is part of the planned path. This will make therapy more targeted and successful as possible. The scalability and generalizability of machine learning models across many populations have become vital, emphasizing the relevance of inclusion in healthcare operations. This is because these models can be applied to many people. Implementing real-time monitoring and feedback mechanisms could change rehabilitation processes. This advances the field greatly. Wearable technologies and continuous data streams can simplify dynamic intervention modifications to meet patients' changing needs. This can be achieved by adopting these technologies. Despite these technological advances, managing the ethical aspects of machine learning in healthcare is crucial. This is because these apps could change patients' lives. To integrate these technologies responsibly and trustworthily, data privacy, consent, and algorithmic bias must be addressed.
